# The neural representation of human *versus* nonhuman bipeds and quadrupeds

**DOI:** 10.1038/s41598-017-14424-7

**Published:** 2017-10-25

**Authors:** Liuba Papeo, Moritz F. Wurm, Nikolaas N. Oosterhof, Alfonso Caramazza

**Affiliations:** 1 Center for Mind/Brain Sciences, University of Trento, Corso Bettini, 31, 38068 Rovereto, TN Italy; 2 CNRS – Institut des Sciences Cognitives Marc Jeannerod – UMR 5304, Univ Lyon, 67 Boulevard Pinel, 69675 Bron, France; 3 Department of Psychology, Harvard University, 33 Kirkland Street, Cambridge, MA 02138 USA

## Abstract

How do humans recognize humans among other creatures? Recent studies suggest that a preference for conspecifics may emerge already in perceptual processing, in regions such as the right posterior superior temporal sulcus (pSTS), implicated in visual perception of biological motion. In the current functional MRI study, participants viewed point-light displays of human and nonhuman creatures moving in their typical bipedal (man and chicken) or quadrupedal mode (crawling-baby and cat). Stronger activity for man and chicken *versus* baby and cat was found in the right pSTS responsive to biological motion. The novel *effect of pedalism* suggests that, if right pSTS contributes to recognizing of conspecifics, it does so by detecting perceptual features (e.g. bipedal motion) that reliably correlate with their appearance. A searchlight multivariate pattern analysis could decode humans and nonhumans across pedalism in the left pSTS and bilateral posterior cingulate cortex. This result implies a categorical human-nonhuman distinction, independent from within-category physical/perceptual variation. Thus, recognizing conspecifics involves visual classification based on perceptual features that most frequently co-occur with humans, such as bipedalism, and retrieval of information that determines category membership above and beyond visual appearance. The current findings show that these processes are at work in separate brain networks.

## Introduction

A requirement for an animal that has evolved to live in a social environment is to recognize entities with which it can engage in social interactions. Humans are very efficient at recognizing conspecifics among other entities in the environment: very early in life, they can visually distinguish conspecifics from other objects^1,2^ and, across the whole life span, they process conspecifics faster and better than other animals^3–5^. How is this tuning for conspecifics realized?

A preference for humans over other biological creatures may emerge already in the visual perceptual system. In various current models, the visual system is tuned for the analysis of socially relevant information; and the recognition of conspecifics through detection of specific perceptual cues is the initial stage that triggers inferential processes culminating in the representation of others’ intentions and mental states^6–10^. An important neural structure for that initial stage would be a region for biological motion perception, in the right posterior superior temporal sulcus (pSTS). Spatially disposed to integrate signals from category-specific regions, the right pSTS has been implicated in forming a representation of human motion and action^11^, and it is regarded as the main entry into the social brain network^6–10^.

Stronger activity in the right pSTS during processing of biological motion *versus* nonbiological motion has been largely documented in neuroimaging studies^12,13^. The relevance of this activity for biological motion perception is supported by research with brain stimulation^14^ and brain-damaged patients^15^. In the current literature, the terms “biological motion” and “nonbiological motion” refer to displays of human motion and object motion, respectively, somehow implying that the visual system encodes all biological motions similarly. Only recently, research has focused on the relationship between human and nonhuman motion in the right pSTS^16^, following reports of behavioural differences in visual perception of moving humans *versus* nonhuman animals.

In particular, it has been shown that, under visual noise, individuals are better at detecting the gait of a man than the gait of a horse, depicted by point light displays (PLDs)^17^. The technique based on PLDs, pioneered by Johansson^18^, is used to depict biological movements by means of few isolated points of light in correspondence with the major joints of the moving body, without visual attributes such as color, form, and texture. The increased tolerance to noise in the case of PLDs of human gait has been taken as evidence of greater visual sensitivity to human (versus nonhuman) motion, by virtue of the human’s higher social value. In keeping with the behavioural difference, stronger right-pSTS activity has been reported during visual perception of PLDs of walking men *versus* walking dogs^19^.

In the current functional MRI (fMRI) study, we investigated whether the stronger response to humans *versus* other familiar biological entities such as dogs, in the biological-motion perception pSTS, reflects a genuine preference for the category of conspecifics or, rather, the effect of perceptual features that differ between a walking man and a walking dog (or a horse). A prominent perceptual difference between a walking man and a walking dog, which could strongly affect a region sensitive to visual motion, is the mode of motion: bipedal *versus* quadrupedal.

It has been shown that the posture in which a body appears – bipedal or quadrupedal – affects the way in which the body is processed. In particular, in a behavioural study, participants exhibited a body-part compatibility effect (faster hand response to vision of upper limbs and faster foot response to lower limbs) comparable to the effect elicited by human bodies, for nonhuman animals in bipedal but not in quadrupedal posture^20^. This finding suggests that nonhuman animals are coded with reference to the human body schema, when presented in a bipedal posture (e.g., a bear standing on its posterior paws). Moreover, it has been shown that visual recognition of biological motion involves an initial stage where the structure of limbs is recovered; this information appears to be necessary to reconstruct, in a second stage, the whole agent^21,22^. This processing of biological motion would be compatible with a recognition-by-parts model^21^, whereby limbs’ structure and motion are particularly diagnostic for visual classification, by virtue of their consistent co-occurrence with a specific category of objects (e.g., bipedalism for prototypical humans).

Following these observations, during fMRI, we presented PLDs of human and nonhuman characters moving in their typical bipedal (man and chicken) or quadrupedal mode (crawling-baby and cat). We also presented scrambled versions of those stimuli, in which dots moved in the same way as in the original PLDs, but their relative positions were varied, so that they formed abstract meaningless shapes. Participants were instructed to report whether two identical video-clips were shown in a row (repetition detection task). We examined whether pSTS responded more strongly to human (i.e., man and crawling-baby) than nonhuman characters (i.e., chicken and cat), irrespective of the within-category variation in the mode of motion. To identify other brain regions sensitive to a perceptually invariant human *versus* nonhuman distinction, we conducted additional multivariate whole brain analyses. Using multivariate pattern analysis (MVPA), we sought to reveal finer-grained neural distinctions captured by the local spatial patterns of activation associated with each character^23,24^.

The results showed stronger right-pSTS activity for bipeds than for quadrupeds. This effect implies that the right pSTS does differentiate among different biological motions, and that this distinction is tied to perceptual features (bipedal *versus* quadrupedal mode of motion), as opposed to more abstract properties that may determine membership to the category of conspecifics. While no brain region showed stronger response to human than nonhuman stimuli (or *vice versa*), a distinction between the two categories, reflected in distinct neural patterns, was found in a network encompassing the bilateral posterior cingulate cortex (PCC) and the left pSTS.

## Methods

### Participants

Twenty healthy volunteers with normal or corrected-to-normal vision participated in the fMRI study (7 female, mean age 26.9 years ± 5.41 *SD*). The Human Research Ethics Committee of the University of Trento approved all procedures, in compliance with the Declaration of Helsinki. Informed consent was obtained from all participants, in written. All methods were performed in accordance with the relevant guidelines and regulations.

### Stimuli and Procedures

Stimuli were 2-s video-clips of point-light displays (PLDs) of four characters walking in their typical manner (lateral view) – a walking man, a walking chicken, a crawling baby and a walking cat – and their scrambled versions (see Supplementary Information for examples of video-clips). Original PLDs were obtained from fully illuminated video-clips modified using Adobe After Effects video processing software (Adobe Systems Inc.), so that the final result was a pattern of 14 moving white dots on a black background. From the original video-clip showing walking cycles of a character for 8–10 s, three fragments of 1.5, 2, or 2.5 s were extracted starting from each of two different frames. The speed of the 1.5-s fragments and of the 2.5-s fragments was accelerated and decelerated, respectively, so that each lasted 2 s. Each video was flipped so that the character faced leftward in six videos and rightward in other six videos. Finally, each video was displayed in 4 different sizes (480 × 360, 393 × 294, 305 × 230, 218 × 164 pixels, corresponding to 100, 140, 180 and 220% of original video-clips, respectively) for a total of 48 versions of a video-clip (2 starting-frames × 3 speeds × 2 orientations × 4 sizes) for each of the four conditions. Scrambled versions of the videos were created, where dots moved in the same way as in the above PLDs but their configuration (i.e., relative positioning) was scrambled, so that the character was no longer recognizable. For each scrambled-PLD, 48 versions were created just like for the PLDs. The variation of speed, size, starting frame and orientation, and the inclusion of scrambled versions of PLDs served to control for the effect on neural activations, of low- and mid-level visual information respectively.

Stimuli were presented during six functional runs (6.64 min each). For every subject, the first three runs included scrambled-PLDs and the last three, PLDs. The fix order was chosen so that the presentation of scrambled-PLDs, introduced as abstract stimuli, was not influenced by prior exposure to PLDs (i.e., to prevent participants from attempting an interpretation of scrambled-PLDs). PLDs were introduced at the end of the third run. In a brief familiarization phase, three video-clips for each character were presented. In this phase, the experimenter named the character in each clip.

Each run comprised: a warm-up phase (10 s), 16 blocks of video-clips (four blocks per category, 12 s each) alternating with 15 baseline phases (12 s each), and a cool-down phase (16 s). In each block, four 2-s video-clips of the same condition were shown, separated by 1 s of fixation. Video-clips of the same condition could vary for speed, size, starting frame or orientation. Participants were instructed to report whether two video-clips, identical with respect to every dimension (i.e., speed, size, etc.), were shown in the same block of four video-clips (repetition detection task). They had to provide the response through yes-or-no keypress at the end of a block, when a question mark appeared on the screen. Stimuli were back-projected onto a screen by a liquid crystal projector (frame rate: 60 Hz; screen resolution: 1024 × 768 pixels). Participants viewed the stimuli binocularly through a mirror above the head coil. The screen was visible as a rectangular aperture of 17.8° × 13°. Presentation of PLDs and scrambled-PLDs did not exceed an aperture of 4.7° × 3.2°. At the end of the scanning section, we checked with debriefing questions, that the participants did not recognize anything/anyone in the first set of video-clips (scrambled-PLDs) and that they could easily recognize each of the four characters in the last set of video-clips (PLDs).

### fMRI data acquisition

Functional images were acquired using echo planar T2-weighted scans (BioSpin MedSpec 4 T Bruker). A total of 1062 volumes of 33 anterior/posterior-commissure aligned slices were acquired over six runs (field of view = 192 × 192 mm; repetition time 2250 ms; echo time 30 ms; flip angle 76°; voxel resolution 3 × 3 × 3 mm^3^; gap 0.45 mm). An additional high-resolution T1-weighted MPRAGE sequence was acquired (176 slices; field of view 256 × 224 mm^2^; repetition time 2700 ms; inversion time 1020 ms, flip angle 7°; voxel resolution 1 × 1 × 1 mm^3^; generalized autocalibrating partially parallel acquisitions with acceleration factor of 2; duration 5.36 min).

### fMRI data preprocessing and analyses

fMRI data pre-processing and statistical analysis were performed with BrainVoyagerQX 2.8.0 (BrainInnovation, Maastricht, NL) and MATLAB (Mathworks Inc., Natick, MA). The first four volumes were discarded prior to image processing. Pre-processing included: spatial realignment and motion correction of the images using the first volume of the first run of PLDs as reference; slice timing correction; removing of low-frequency drifts with a temporal high-pass filter (cutoff frequency 3 cycles per run); spatial smoothing with a Gaussian kernel of 8-mm FWHM for univariate analyses, and of 3-mm FWHM for multivariate analyses; and transformation of data into Talairach space. Subject-specific β-weights were derived through a general linear model (GLM) including the eight conditions (PLDs and scrambled-PLDs of human and nonhuman bipeds and quadrupeds) and the question as regressors of interest and six motion parameters as regressors of no interest. All regressors were convolved with a standard hemodynamic response function. Multivariate analysis (MVPA with cross-validated classification) was performed using the CoSMoMVPA Toolbox^25^ through MATLAB. In the following analyses, statistical significance for second-level (group) analyses was determined using an initial voxelwise threshold of *P* < 0.001 and a cluster-level threshold of *P* = 0.05 using Monte Carlo simulations (10,000 iterations), as implemented in the CoSMoMVPA Toolbox.

### fMRI univariate analyses

We used the whole-brain contrast PLDs > scrambled-PLDs to identify the bilateral pSTS region responsive to biological motion (right: 44, −68, 15; left: −49, −74, 24; *P* < 0.001, uncorrected; for other clusters from the same contrast, see Table 1). To define the individual region-of-interest (ROI) in pSTS, we performed the same contrast for each individual’s GLM, identified the peak activity (*P*s ≤ 0.05, uncorrected) within 20 mm from the group-average activity-peak along the three axes (x, y and z), and created a sphere of 6-mm around the individual peak coordinates (see Supplementary Table S1 for individual peak coordinates). From the right and left ROIs of each participant, we extracted the β-weights relative to each condition (man, chicken, baby and cat) and subtracted the activity (β-weights) relative to the corresponding scrambled video-clips to minimize the differences across conditions due to lower- and mid-level visual features. Two separate repeated-measures ANOVAs were performed for the left and right pSTS-ROI, respectively, with the factors Pedalism (bipedal, quadrupedal) and Category (human, nonhuman).

**Table 1:** Location and significance of clusters showing stronger activity for PLDs relative to scrambled-PLDs. The results are cluster-based corrected using Monte Carlo simulations (10.000 iterations).

		X	Y	Z	*t*	*p*
Right	Anterior middle temporal gyrus	41	13	−24	4.90	0.000098
	Parahippocampal gyrus	23	−14	−12	6.36	0.000004
	Lingual gyrus	−25	−17	−15	7.74	0.000000
Left	Posterior cingulate cortex	2	−20	36	6.82	0.000002
	Medial prefrontal cortex	−1	43	−6	9.50	0.000000
	Inferior frontal gyrus	−34	31	−6	5.55	0.000024

### MVPA with cross-validated classification

In order to increase sensitivity to the neural distinction between human and nonhuman stimuli, we used MVPA with linear support vector machine (SVM) classifiers^26^ (LIBSVM, http://www.csie.ntu.edu.tw/~cjlin/libsvm), to obtain the neural patterns of response to each of the experimental stimuli, and test the similarity among them^27^. In this analysis, for each participant, 12 multivariate β-patterns per condition (one for each of four blocks per run) were estimated in each 3-voxel-radius sphere centred in each voxel in the brain (searchlight MVPA). A classifier was trained and tested on individual subject data, using a leave-one-out cross-validation strategy. In the current design, a region that distinguishes between humans and nonhumans should show distinct neural patterns for all the pairs that instantiate that distinction: man *versus* cat, man *versus* chicken, baby *versus* cat and baby *versus* chicken. This was tested as follows. In the first cross-validation scheme, the classifier was trained on 22 patterns (11 from the man- and 11 for chicken-specific patterns) and tested on its accuracy at classifying two patterns from the baby- and the cat-condition, and *vice versa* (i.e., training on baby *versus* cat and test on man *versus* chicken). This procedure was performed in 24 iterations. Classification accuracies from all iterations were averaged to give the mean classification accuracy for each participant. The same approach was repeated in a second cross-validation scheme, where the classifier was trained on man *versus* cat and tested on baby *versus* chicken, and *vice versa* (i.e., training on baby *versus* chicken and test on man *versus* cat).

For each classification scheme, classification accuracies were summarized at the centre voxel (“summary voxel”) of each sphere of the searchlight, yielding maps of classification accuracy values. Individual maps were averaged to obtain mean accuracy maps. In addition, individual maps were entered into a one-sample *t* tests, separately for each scheme. A conjunction of the two statistical maps was computed based on the lower common *t* value per voxel to identify the brain structures with above-chance classification accuracy for both schemes^28^.

To test whether putative effects could be due to low-level visual characteristics of the PLDs like single dot movement trajectories, speed, or accelerations, we performed the same classification procedure using the scrambled versions of the PLDs. For the classification of scrambled PLDs we computed mean accuracy and statistical maps. In addition, we subtracted the scrambled from the nonscrambled maps for each participant.

In sum, to emphasize perceptually invariant human-nonhuman distinction and reduce the decoding of merely perceptual information, we implemented two strategies. First, we increased the variability within each condition by varying speed, size, gait direction (leftward or rightward) and starting-frame. Second, we included two different instances for each relevant condition (i.e., baby and man for humans, and chicken and cat for nonhumans). The analysis on scrambled PLDs provided further control for the effect of human-nonhuman discrimination.

The dataset generated and analysed during the current study is available from the corresponding author on request.

## Results

### Behavioural data

Participants performed better with PLDs than with scrambled-PLDs (RTs: *F*(19) = 6.003, *P* = 0.024; accuracy: *F*(19) = 21.140, *P* < 0.001) although the accuracy was high for both types of stimuli (means: 86% PLDs; 81% scrambled-PLDs). Accuracy rates and RTs were comparable across the four critical conditions (man, chicken, baby and cat; all *P*s ≥ 0.309).

### fMRI data

#### Representation of humans and nonhumans in the biological-motion processing pSTS

**Figure 1:**
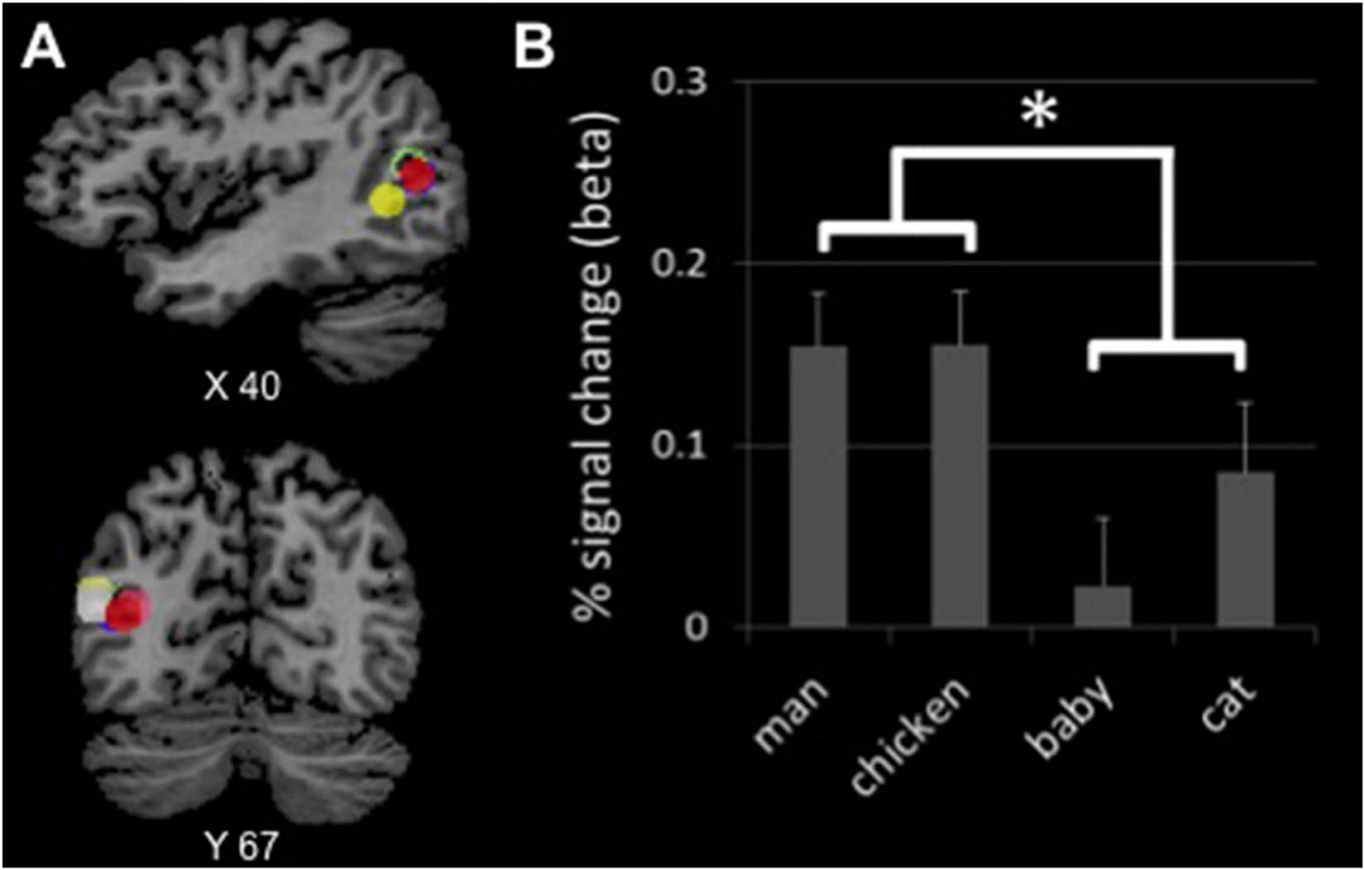
Increased activity in the right pSTS for bipeds (man and chicken) relative to quadrupeds (crawling-baby and cat). (**A**) Regions of interest centred in the right pSTS peaks, defined with the contrast PLDs > scrambled-PLDs. Different colours correspond to different subjects. (**B**) β-weights for PLDs of human and nonhuman bipeds and quadrupeds in the right pSTS. Error bars denote within-subject standard errors of the mean. * denotes statistically significant effect (*P* < 0.05).

The analysis of variance with factors Pedalism (bipedal and quadrupedal) and Category (human and nonhuman) identified an effect of Pedalism in the right pSTS, *F*(1,19) = 7.759, *P* = 0.012, with greater activity for bipeds than quadrupeds, but no effect of Category, *F*(1,19) = 0.746, *P* = 0.398, or interaction, *F*(1,19) = 0.470, *P* = 0.501 (Fig. 1). Activations in left pSTS showed no significant effect, although there was a trend for the effect of Pedalism, congruent with the effect found in right pSTS (Pedalism, *F*(1,19) = 3.190, *P* = 0.090; Category, *F*(1,19) = 0.128, *P* = 0.724; interaction, *F*(1,19) = 0.348, *P* = 0.562).

No stronger activity for humans over nonhumans was found in the ROI analysis focusing on pSTS or in the univariate whole-brain analysis. This motivated further investigation with multivariate analyses.

#### The human-nonhuman distinction throughout the brain

**Figure 2:**
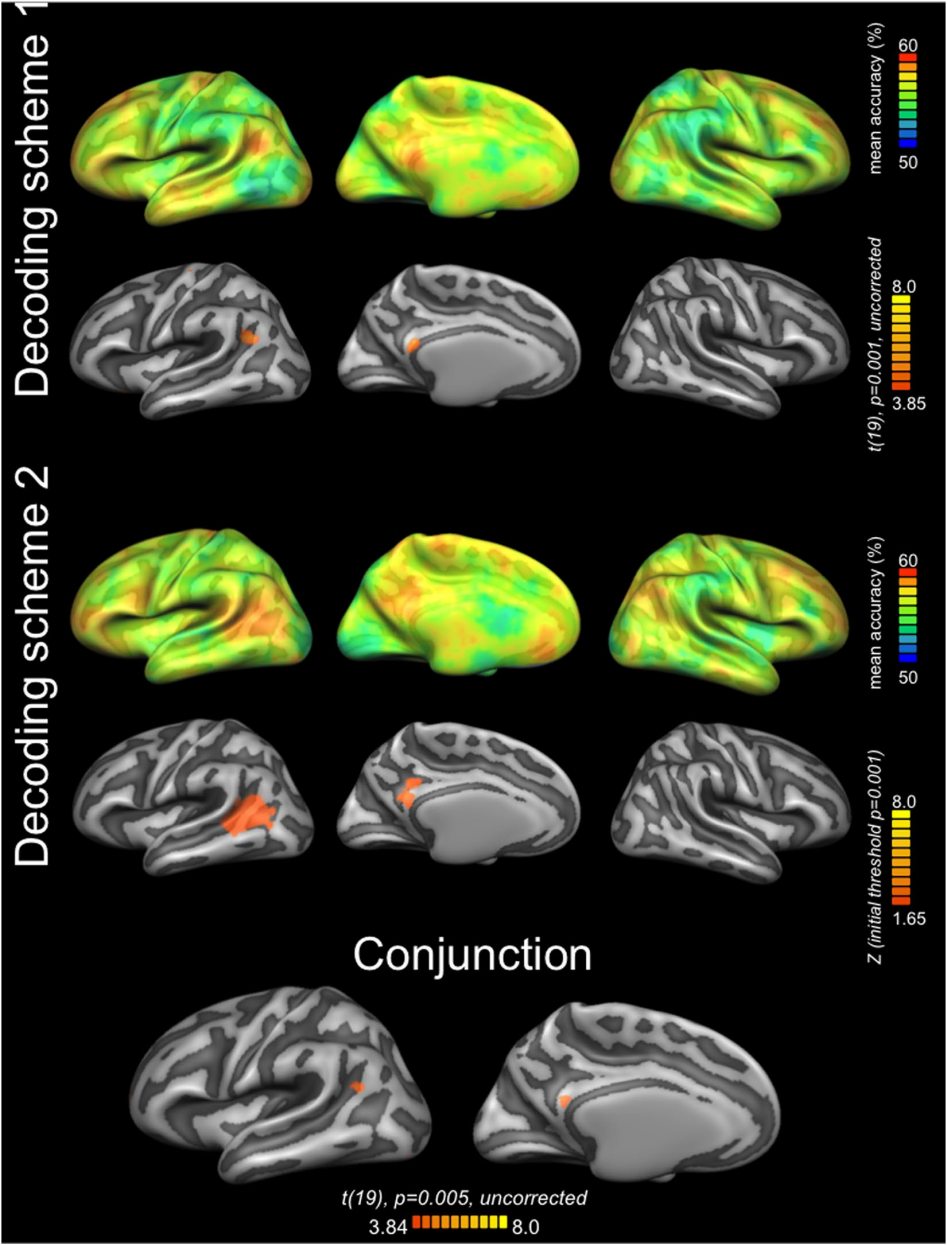
Brain network for the human-nonhuman distinction. For each decoding scheme, the upper row shows the mean accuracy map obtained from individual accuracy maps, cortex-based aligned, averaged, and projected onto a common group surface (decoding accuracy at chance is 50%). For each decoding scheme, the lower row shows the statistical map obtained by entering the individual maps into a one-sample *t* tests. The conjunction of the two statistical maps shows the brain structures with above-chance classification accuracy for both schemes. Decoding scheme 1: training on man *versus* chicken, test on baby *versus* cat, and *vice versa*; Decoding scheme 2: training on man *versus* cat, test on baby *versus* chicken, and *vice versa*. Lateral views of the left and right hemisphere are shown in the first and third column respectively; ventral sagittal view of the left hemisphere is shown in the medial column.

As reported above, we found no univariate effect of Category (i.e., stronger activity for humans than nonhuman, or *vice versa*) in the ROIs or across the whole brain. It is therefore possible that human and nonhuman representations are encoded in one and the same brain region, but in distinct subpopulations of that common region. Using searchlight MVPA, we aimed at identifying brain regions that are sensitive to the categorical human-nonhuman distinction, independently from the mode of biological motion that characterizes a character (bipedal or quadrupedal). With the two cross-validation schemes described above, we decoded humans vs. nonhumans across pedalism, reasoning that a brain region that differentiates between human and nonhumans should show significant above chance accuracies in both schemes. For both schemes, we found effects overlapping in left pSTS and in left and right PCC (Fig. 2 and Table 2). While only for the second scheme the effects in both regions survived the correction for multiple comparisons, classification accuracies were not statistically different between the two decoding schemes. In particular, a whole-brain contrast using the statistical maps obtained from the two decoding schemes showed no significant clusters in the left pSTS and the PCC, with a voxelwise threshold of *P* = 0.001, or more liberal thresholds of *P* = 0.005 and *P* = 0.01.

**Table 2:** Clusters for human-nonhuman distinction identified with the searchlight MVPA using a cross-validation approach for decoding scheme 1 and 2 (chance accuracy is 50%) and the conjunction of the maps obtained by the two schemes.

		Cluster				Peak					
		*t*	*p*	accuracy	size	*t*	*p*	accuracy	x	y	z
***Decoding Scheme 1 (train: man vs***. ***chicken***, ***test: baby vs***. ***cat)***											
L	pSTS	4.185	0.0005*	56.7	251	5.287	0.00004	57.9	−45	−52	22
L	PCC	4.328	0.0004*	56.5	359	5.576	0.00002	57.7	−9	−46	16
R	IOG	4.43	0.0003*	58	484	5.757	0.00001	60.7	12	−82	−17
L	IOG	4.258	0.0005*	56.9	2257	5.578	0.00002	58.3	−16	−100	−8
***Decoding Scheme 2 (train: man vs***. ***cat test: baby vs***. ***chicken)***											
L	pSTS	4.351	0.0005	56	1825	5.829	0.00001	58.3	−48	−45	13
L	PCC	3.978	0.0008	56.2	667	4.675	0.00014	58	−6	−46	22
***Conjunction***											
L	pSTS	3.411	0.0031	55.9	69	4.264	0.00038	56.9	−48	−58	19
L	PCC	3.393	0.0031	55.4	202	3.864	0.00097	56	−12	−43	22

Statistical maps of classification accuracy in %; size in mm^3^. The results of Decoding Scheme 2 are cluster-based corrected using Monte Carlo simulations (10.000 iterations). The results of Decoding Scheme 1 are uncorrected. *Values uncorrected at cluster level; R, right; L, left; PCC, posterior cingulate cortex; IOG, inferior occipital gyrus; pSTS, posterior superior temporal sulcus.

The same analysis using the scrambled version of the four PLD types revealed that for both schemes classification accuracies in left pSTS and PCC were at chance and no other region revealed significant above chance accuracies (Figure S1 of Supplementary Information). This suggests that low-level features of PLDs, which were retained in the scrambled videos (i.e., movement trajectories or the speed/acceleration profiles of points), did not account for the decoding of humans versus nonhumans.

## Discussion

How do humans recognize conspecifics? We interpreted this fundamental question as asking what it means to recognize humans. This process could involve processing of perceptual features characteristic of conspecifics and/or the retrieval of more abstract information that determines category membership (human or nonhuman). Here, we identified both processes, at work in separate brain networks, contributing to distinguishing between humans and nonhumans. In particular, we found evidence for detection of perceptual features in right pSTS and category membership assignment in left pSTS and bilateral PCC.

Reports of higher activity for humans, relative to familiar, nonhuman animals such as dogs, in the circuitry for biological-motion perception centred in the right pSTS^18^, have suggested that the human perceptual system is tuned to conspecifics possibly because of their highest social value^7–10^. Stronger right pSTS activity for moving humans *versus* moving nonhuman characters has suggested sensitivity to differences within the large category of biological motion. However, that effect can reflect a tuning for the category of conspecifics, as well as a tuning for visual perceptual features that are characteristic of the typical conspecific, but not of biological entities such as dogs and horses. The current results speak to this question.

In keeping with previous findings, we found that the right pSTS, functionally localized during biological motion perception, showed stronger response to PLDs of a human adult (i.e., a man) than to cat-PLDs. However, the right-pSTS activity was also stronger for chicken-PLDs than for baby-PLDs and comparable for chicken-PLDs and man-PLDs (and for baby- and cat-PLDs). This pattern of response revealed the novel *effect of pedalism*, whereby the two instances of biological bipedal motion in the current design (walking man and walking chicken) elicited stronger right-pSTS activity relative to two instances of biological quadrupedal motion (crawling-baby and walking cat). This effect suggests representation of a perceptual feature, bipedalism, which is statistically associated with the prototypical human adult, as opposed to representation of the abstract category of humans.

Anecdotal reports of PLDs of bipedal animals being misclassified as humans^29,30^ have suggested that bipedal motion is an important perceptual cue for recognizing conspecifics. Moreover, retrieving the structure of limbs has been described as a critical stage in the process that leads to recognition of a biological entity from its motion^22,30,31^. This is compatible with a recognition-by-parts model^21^, involving an initial stage where the structure of limbs is recovered from the local motion signals, and a second stage where information about limbs is used to reconstruct the whole agent^22^. In this model, limbs are defined as features of intermediate complexity^32^, which are particularly informative for visual classification because of their systematic co-occurrence with a specific category of objects (e.g., bipedalism for prototypical humans). In this framework, our results show that the human visual system attaches specific significance to limbs, in that limbs define a feature (i.e., the mode of motion) that can guide visual classification within the large variety of biological entities. Differential activity for bipedal *versus* quadrupedal motion in the biological motion-perception right pSTS implies sensitivity to distinctions based on visual features.

The tuning for bipedal motion in the right pSTS could reflect the pressure of our phylogenetic and/or ontogenetic history on the perceptual systems for recognizing conspecifics, although, at this point in the brain network, there is not (yet) a representation of, or a preference for, the abstract category of conspecifics or the encoding of human-nonhuman distinction.

Such distinction is computed outside the biological motion-perception pSTS. Despite physical dissimilarity, the neural patterns for man- and baby-PLDs were consistently classified together, and as distinct from the patterns for chicken- and cat-PLDs, in a network encompassing the bilateral PCC and the left pSTS. Neural patterns in those regions distinguished between humans and nonhumans across all instances that represented that distinction in the current design (man *versus* cat, man *versus* chicken, baby *versus* cat and baby *versus* chicken). Moreover, given the demand to attend to low-level features of the stimuli (size, movement trajectories, or speed of PLDs) for repetition detection, the access to non-perceptual information that determines the human-nonhuman distinction occurred automatically.

The current findings add to previous fMRI research addressing the cortical representation of object-categories above and beyond distinctions based on physical/perceptual properties or on input modality. This research has shown that neural responses to objects in the PCC and posterior temporal cortex respect the supramodal criterion (i.e., they are independent from the modality through which objects are presented), suggesting abstract representation of semantic properties^33^.

We shall highlight that the human-nonhuman distinction survived the cluster-level correction for one of the two classification schemes only. Thus, the current results should be treated with caution. However, at the very least, they emphasize neural representations of biological entities based on dimensions, independent of low-level features such as movement trajectories and speed/acceleration profiles (see analyses of scrambled stimuli), and independent of – and more abstract than – the type of biological motion (bipedal or quadrupedal) seen in the right pSTS.

The PCC has been proved selective to the representation of people knowledge^34–36^; while the posterior middle/superior temporal cortex has been implicated in the representation of abstract properties of motion and action^37–39^. The present study suggests that information in both regions contributes to determine membership in the category of conspecifics/humans above and beyond their physical appearance. Further research should address the specific information that determines the human-nonhuman distinction in left pSTS and PCC. That information could be highlighted more strongly in task settings in which processing of higher-level properties of the stimuli is less shallow than it was in the current study, where participants’ attention was driven toward low-level visual features of the stimuli.

Other brain sites may contribute to characterize humans. For example, the right dorsolateral prefrontal cortex has been shown to discriminate between the face of a man *versus* the face of a dog or a monkey^40^. Different stimuli (static faces *versus* point-light displays) may emphasize different aspects of biological characters, yielding different results. A critical element may be the inclusion of the baby-condition in the current design, which was not present in the previous one. This novel condition may have been crucial to isolated neural information that assigns man and baby to the same taxonomic category, irrespective of perceptual differences or differences in abstract, psychological features between the prototypical human (the adult human) and every other creature, including the baby. For example, dimensions of mind perception, such as agency and the ability to think, plan and work toward goals, are assigned to a uniquely maximal degree to the adult human, and to various but lesser degrees to other biological creatures such as baby, cat, chicken, monkey and dog^41^.

In conclusion, recognition of conspecifics is achieved through the collective activity of separate brain networks to detect the perceptual/physical features that reliably correlate with the appearance of a conspecific (e.g., bipedal motion in the right pSTS), and to retrieve abstract information that determines species membership (i.e., human-nonhuman in the left pSTS and PCC).

## Electronic supplementary material

Supplementary Information

Supplementary video 1

Supplementary video 2

Supplementary video 3

Supplementary video 4

Supplementary video 5

Supplementary video 6

Supplementary video 7

Supplementary video 8
