# Supplementary material for: The neural representation of human versus nonhuman bipeds and quadrupeds
Source: Sci Rep. 2017 Oct 25;7:14040. doi: 10.1038/s41598-017-14424-7 (PMC5656636; doi:10.1038/s41598-017-14424-7)
Supplement: Supplementary file 1 — Supplementary Information [file 41598_2017_14424_MOESM1_ESM.pdf]

Supplementary information

The neural representation of human *versus* nonhuman bipeds and quadrupeds

Liuba Papeo[a,b,1], Moritz F. Wurm[a,c], Nikolaas N. Oosterhof[a], & Alfonso Caramazza[a,c]

[a]Center for Mind/Brain Sciences, University of Trento, Corso Bettini, 31, 38068 Rovereto TN, Italy; [b]CNRS – Institut des Sciences Cognitives Marc Jeannerod – UMR 5304 , Univ Lyon, 67 Boulevard Pinel, 69675 Bron, France; [c]Department of Psychology, Harvard University, 33 Kirkland Street, Cambridge MA 02138, USA

[1]Corresponding author: CNRS – Institut des Sciences Cognitives Marc Jeannerod – UMR 5304, Univ Lyon, 67 Boulevard Pinel, 69675 Bron, France, Email: liuba.papeo@gmail.com

Table S1. Individual peak coordinates used to define ROIs in the bilateral pSTS.

	rigth pSTS			left pSTS		
	x	y	z	x	y	z
Sub_01	42	-68	15	-43	-65	16
Sub_02	39	-65	15	-43	-68	19
Sub_03	47	-77	12	-45	-71	19
Sub_04	38	-67	12	-43	-65	15
Sub_05	53	-65	6	-50	-74	18
Sub_06	41	-68	12	-49	-65	12
Sub_07	47	-77	12	-43	-68	18
Sub_08	47	-77	12	-46	-73	6
Sub_09	44	-59	4	-43	-65	15
Sub_10	47	-65	18	-49	-64	25
Sub_11	39	-66	15	-43	-66	15
Sub_12	36	-65	14	-43	-65	15
Sub_13	35	-65	14	-43	-69	18
Sub_14	47	-65	15	-43	-65	15
Sub_15	36	-65	15	-49	-65	13
Sub_16	47	-77	12	-47	-74	6
Sub_17	50	-77	12	-46	-69	17
Sub_18	35	-65	14	-49	-78	23
Sub_19	47	-77	12	-40	-74	8
Sub_20	38	-68	12	-43	-65	15

Supplementary Figure 1

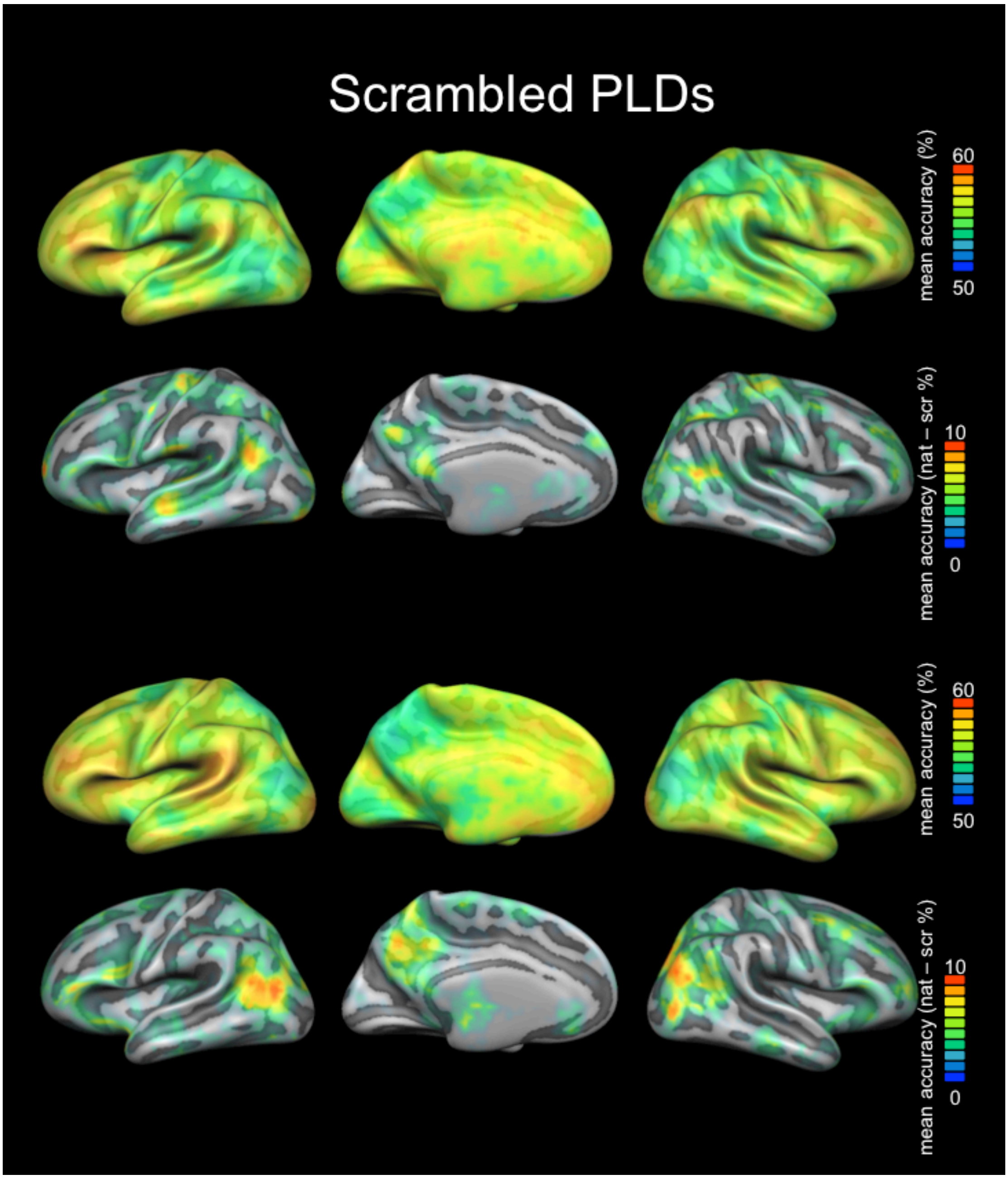

Figure S1. Results of the MVPA with a cross-validation approach for two decoding schemes using the scrambled versions of the four PLD types. Decoding scheme 1: training on scrambled_man versus scrambled_chicken, test on scrambled_baby versus scrambled_cat, and *vice versa*; decoding scheme 2: training on scrambled_man versus scrambled_cat, test on scrambled_baby versus scrambled_chicken, and *vice versa*. For both decoding schemes, the upper row shows the mean accuracy map obtained

from individual accuracy maps, cortex-based aligned, averaged, and projected onto a common group surface (decoding accuracy at chance is 50%). For both decoding schemes, the lower row shows the mean accuracy map representing the difference between by the decoding accuracy for natural PLDs (nat) and the decoding accuracy for scrambled PLDs (scr). Decoding scheme 1: training on man versus chicken, test on baby versus cat, and vice versa; decoding scheme 2: training on man versus cat, test on baby versus chicken, and vice versa. Lateral views of the left and right hemisphere are shown in the first and third column respectively; ventral sagittal view of the left hemisphere is shown in the second column. Lateral views of the left and right hemisphere are shown in the first and third column respectively; ventral sagittal view of the left hemisphere is shown in the medial column.
